# Impact of Palliative Care Consultation Service on Terminally Ill Cancer Patients: A 9-Year Observational Cohort Study in Taiwan

**DOI:** 10.1097/MD.0000000000002981

**Published:** 2016-03-11

**Authors:** Ching-Yi Lu, Wen-Chi Shen, Chen-Yi Kao, Hung-Ming Wang, Shu-Chuan Tang, Tsu-Ling Chin, Chuan-Chuan Chi, Jin-Mei Yang, Chih-Wen Chang, Ying-Fen Lai, Ya-Chi Yeh, Yu-Shin Hung, Wen-Chi Chou

**Affiliations:** From the Department of Nursing (C-YL, S-CT, T-LC, C-CC, J-MY, C-WC, Y-FL, Y-CY), Division of Hematology-Oncology, Department of Internal Medicine, Chang Gung Memorial Hospital at Linkou (W-CS, C-YK, H-MW, Y-SH, W-CC), and Graduate Institute of Clinical Medical Sciences, College of Medicine, Chang Gung University, Taoyuan, Taiwan (W-CC).

## Abstract

The palliative care consultation service (PCCS) that has been enthusiastically promoted in Taiwan since 2005 was designed to provide comprehensive end-of-life care for terminally ill patients with qualified interdisciplinary specialists in acute care ward setting. This study aims to evaluate the impact of PCCS on terminally ill cancer patients.

A total of 10,594 terminal cancer patients who were referred to PCCS from a single medical center in Taiwan between 2006 and 2014 were enrolled. The percentages of patients’ and their families’ disease awareness, do-not-resuscitate (DNR) designation, refusal and acceptance of palliative care among terminally ill cancer patients were analyzed retrospectively.

At the beginning of PCCS, the percentages of disease awareness among patients and their family were increased from 25.4% to 37.9% (*P* = 0.007) and from 61.2% to 84.7% between 2006 and 2014 (*P* = 0.001), respectively. Patients’ disease awareness after PCCS referral between 2006 and 2014 was increased from 47.1% to 64.5% (*P* = 0.016). Family's awareness of diagnosis and prognosis after PCCS referral researched to a steady plateau, 94.1% to 97.8% in different year cohort (*P* = 0.34). The percentage of DNR designation rate at the beginning of PCCS (in 2006) was 15.5%, and the designation rate was increased annually and finally reached to 42.0% in 2014 (*P* = 0.004). The percentage of DNR consents after PCCS was also improved from 44.0% in 2006 up to 80.0% in 2014 (*P* = 0.005). PCCS refusal rate decreased gradually and dropped to 1.6% in 2014 (*P* = 0.005). The percentage of PCCS utilization was increased 5-fold during the 9-year period after the promotion of PCCS

In the program of PCCS promotion, an increasing trend of PCCS utilization, better patients’ and their families’ awareness of diagnosis and prognosis, more consent to DNR, more patients were discharged with stable condition at the end of PCCS and a decrease refusal rate of end-of-life palliative care among terminal cancer patients were observed in Taiwan between 2006 and 2014.

## INTRODUCTION

Cancer-related mortality is the leading cause of death in Taiwan since 1982 while national healthy statistic was available, and it accounts for around 25% of total cause of death annually.^1^ Since a large population has died of cancer, palliative care has been promoted in Taiwan for more than 2 decades in order to improve quality of end-of-life care among terminally ill cancer patients. Moreover, palliative care was supported after the enactment of the Nature Death Act in Taiwan was legislated in 2000.

Palliative care is a patient- and family-centered treatment designed to relieve terminal illness-induced symptoms, and to optimize patients’ and their family's quality of life by anticipating and reducing sufferings.^2^ Therefore, palliative care has been implemented in cancer patients for decades in Western countries.^2^ However, according to the analysis of national health insurance database in Taiwan, the acceptance of palliative care among terminally ill cancer patients in the past few years (2001–2006) increased limitedly (from 11% to 17%).^3^ Limited hospice resource was one of the major reasons for slow growth in hospice utilization.^3–5^ In addition, patients’ lack of awareness of their diseases and prognoses,^6,7^ philosophies of end-of-life and misinterpretations about the functions of hospice units (places for dying) also delayed growth of hospice use.

The palliative care consultation service (PCCS) that has been enthusiastically promoted in Taiwan since 2005 was designed to provide comprehensive end-of-life care for terminally ill patients with qualified interdisciplinary specialists in acute care ward setting.^8–10^ This study aims to evaluate the impact of PCCS on terminally ill cancer patients by observing a single but the largest medical center's experience in Taiwan between 2006 and 2014.

## MATERIALS AND METHODS

### Patient Selection

Terminal cancer patients who were admitted to Chang Gung Memorial Hospital, Linkou Branch and were referred to PCCS from January 2006 to December 2014 were enrolled. All of the patients who were referred to PCCS had either pathological- or radiography-proven malignancies, and could benefit from PCCS and were unlikely to survive more than 6 months based on their clinicians’ judgments. Patients and/or families who refused care by PCCS were excluded. Patient characteristics, the percentages of patients’ and their families’ disease awareness, do-not-resuscitate (DNR) designation, refusal and acceptance of palliative care among terminally ill cancer patients after care from the PCCS were analyzed retrospectively. The study protocol was approved by the Institutional Review Board of the hospital.

### Palliative Care Consultation Service (PCCS) Setting

A multidisciplinary palliative care team consists qualified palliative care/hospice physicians, nurse specialists, social workers, psychologists, and a religious worker (Buddhist). All patients received joint care from their primary care physician and care of PCCS, aiming to improve end-of-life quality with symptoms controls and holistic care to both the patient and family. After signing an informed consent, a patient was visited by the team physician and nurse and followed on a weekly basis thereafter. Immediate feedback to the patient's primary care staff after each PCCS consultation was highly recommended. Services from other team members were provided upon requests from the physician or the nurse. For patients unaware of their own conditions, the PCCS strive for helping them understanding/acknowledging their conditions, assisting in signing DNR consents and planning for end-of-life wills. PCCS was discontinued when the patient, (1) died, (2) transferred to acute palliative care or home care, (3) discharged from the hospital under stable condition, or (4) no longer required PCCS.

### Data Collection

The team registered nurse/nurse specialist/nurse practitioner completed an electronic case record^11,12^ and evaluated disease awareness of both the patient and family.^13^ The PCCS utilization rate was calculated by dividing the number of annual consultations by total cancer patients died in our institution each year. The cancer related deaths were confirmed from either institutional cancer registry center or the National Register of Death Database in Taiwan.

### Statistical Analysis

Basic demographic data were summarized as n (%) for categorical variables and mean values for continuous variables, respectively. Statistically changes of disease awareness, difference of disease awareness between the patients and their family, DNR designation, and PCCS utilization, as well as patients’ outcomes at the end of PCCS over the study period were examined by using Cochran–Armitage test.^14^ Statistical analyses were performed by using SPSS 17.0 statistical software (SPSS, Inc, Chicago, IL). All statistical assessments were 2-sided. A *P*-value small then 0.05 was considered as significant.

## RESULTS

Total 10,594 terminally ill cancer patients referred to PCCS between 2006 and 2014 were enrolled in this study. The number of referred patients increased from 232 patients to 1965 patients per year (Table 1). The mean age of patients varied from 51 to 64 years, and the proportion of male gender was 55% to 62% in different year cohort. The 5 leading referral departments were oncology, other medical, surgery, pediatrics, and emergency. Patients referred from emergency department were significantly increased in this period. Moreover, total number of referred patients from all departments to PCCS was increased annually between 2006 and 2014.

**TABLE 1:**
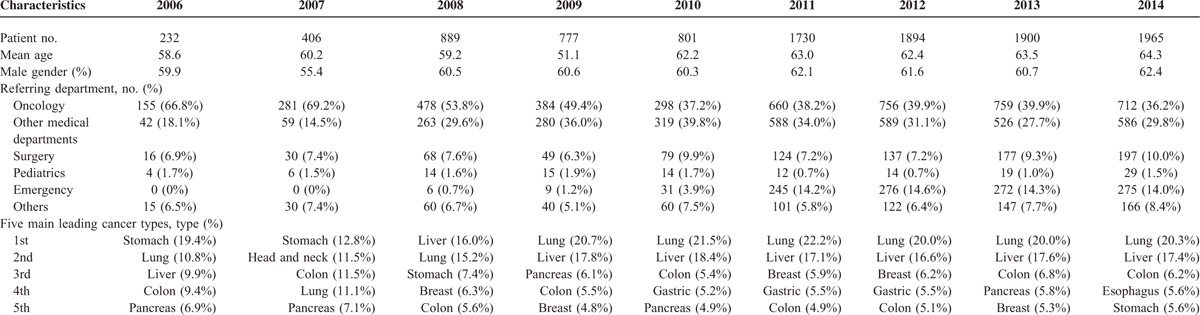
Basic Demographic Data of Terminally Ill Cancer Patients Referred to Palliative Care Consultation Service Between 2006 and 2014

The leading cancer type among PCCS referrals was gastric cancer in 2006 and 2007, but the percentage of gastric cancer in total referred patients started to decrease in 2008, and then remained consistent (Table 1). The number of patients with lung and liver cancers increased dramatically from 2006 to 2014, and became major dominant cancer types since 2008. The order of cancer types among patients referred to PCCS was similar to the sequence in cancer-related deaths in Taiwan since 2008.^1^

**FIGURE 1:**
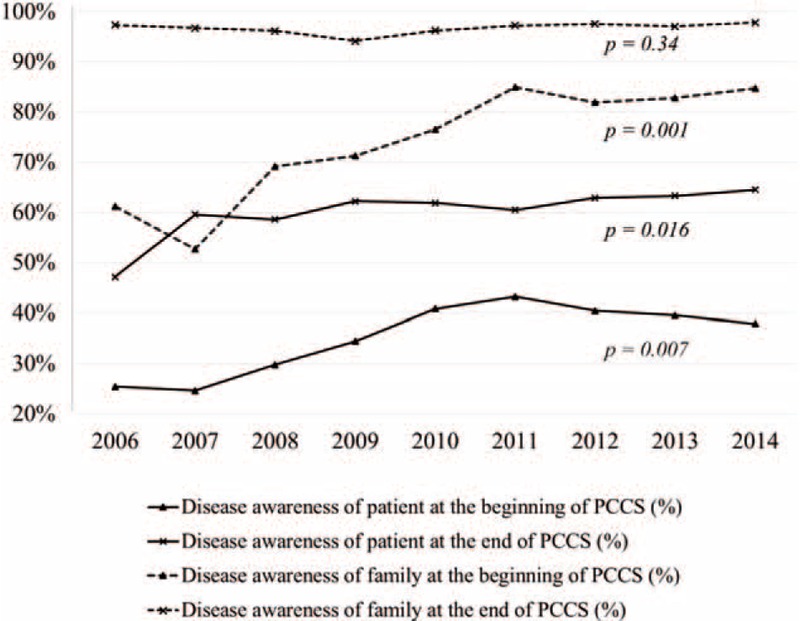
Terminally ill cancer patients’ and their family's disease awareness at the beginning and the end of palliative care consultation service between 2006 and 2014.

The percentages of disease awareness as well as DNR designation at the beginning and the end of PCCS are shown in Table 2. At the beginning of PCCS, the percentages of disease awareness among patients and their family were increased from 25.4% to 37.9% (*P* = 0.007) and from 61.2% to 84.7% between 2006 and 2014 (*P* = 0.001), respectively. The percentage of DNR at the beginning of PCCS (in 2006) was 15.5%, and the acceptance rate was increased annually and finally reached to 42.0% in 2014 (*P* = 0.004). Patients’ disease awareness after PCCS referral between 2006 and 2014 was increased from 47.1% to 64.5% (*P* = 0.016). Family's awareness of diagnosis and prognosis after PCCS referral researched to a steady plateau, 94.1% to 97.8% in different year cohort (*P* = 0.34). The percentage of DNR consents after PCCS was also improved from 44.0% in 2006 up to 80.0% in 2014 (*P* = 0.005). Figure 1 shows the upward trends of disease awareness among patients and their family at the beginning and the end of PCCS in terminally ill cancer patients over the study period.

**TABLE 2:**
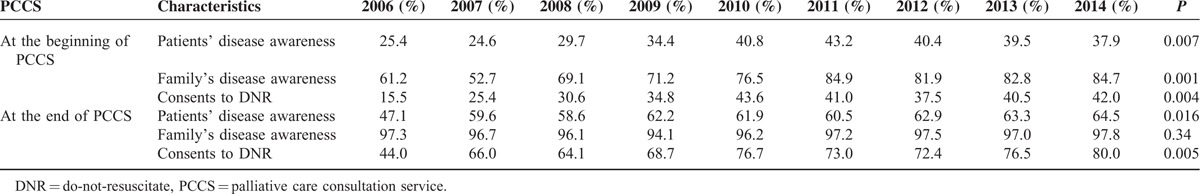
Percentage of Disease Awareness Among Patients/Family and Their DNR Acceptance Rate at the Beginning and the End of Care With Palliative Care Consultation Service (PCCS) Between 2006 and 2014

**FIGURE 2:**
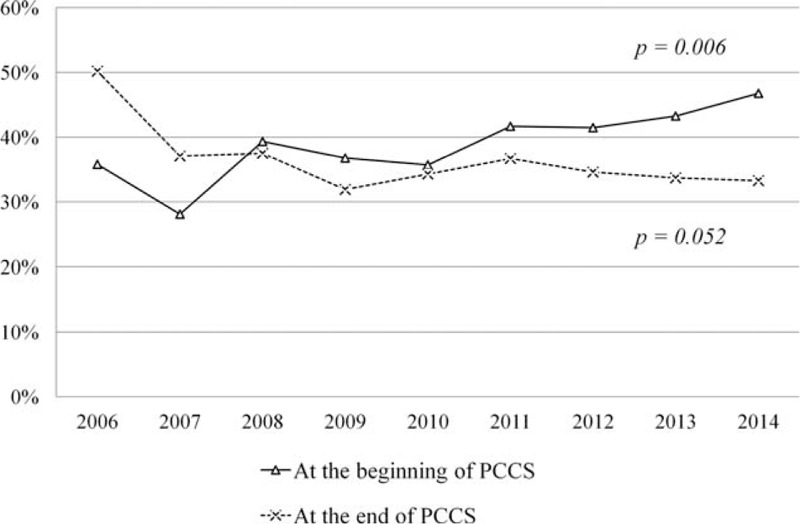
Percentage of differences in disease awareness between terminally ill cancer patients and their family at the beginning (solid line) and the end (dash line) of the palliative care consultation service between 2006 and 2014.

The differences of disease awareness between patients and their family at the beginning and the end of PCCS over the study period are shown in Figure 2. Family was always more aware of the disese than patients themselves during the study period. At the beginning of PCCS, the differences of disease awareness between patients and their family were increased from 35.8% in 2006 to 46.8% in 2014 (*P* = 0.006). At the end of PCCS, the gaps of disease awareness between patients and their family were decreased from 50.2% in 2006 to 33.3% in 2014 (*P* = 0.052).

**FIGURE 3:**
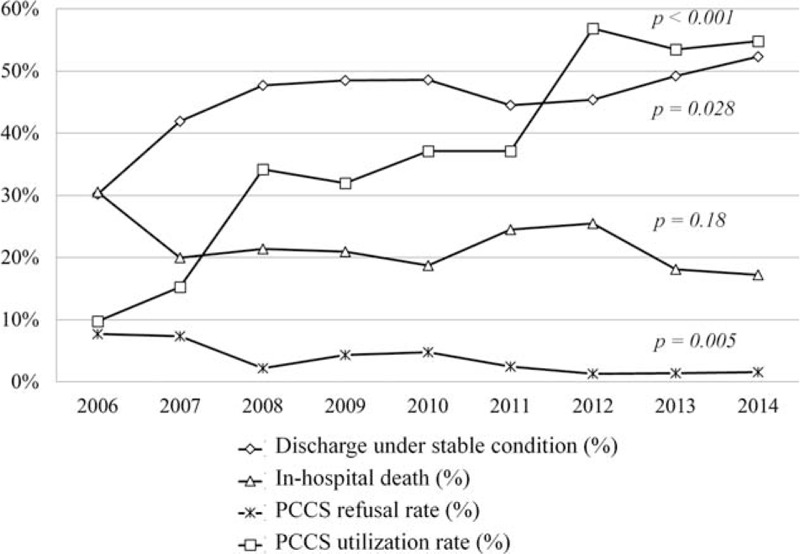
Patient's outcome after palliative care consultation service (PCCS)—PCCS utilization and refusal rate in terminally ill cancer patients between 2006 and 2014.

Patient's outcome at the end of PCCS is shown in Table 3 and Figure 3. Eight percent of patients refused to receive PCCS care in the first year of PCCS promotion in 2006. PCCS refusal rate decreased gradually and dropped to 1.6% in 2014 (*P* = 0.005). The percentage of discharge under stable condition after PCCS care was increased from 30.2% to 52.3% between 2006 and 2014 (*P* = 0.028), and the percentage of in-hospital death after PCCS care was decreased from 30.6% to 17.2% within the same study period (*P* = 0.18). The number of cancer-related death varied from 1721 to 2011 patients in different year cohort between 2006 and 2014 in our institute. Among these patients, PCCS utilization rate increased dramatically from 9.8% to 54.8% between 2006 and 2014 (*P* < 0.001).

**TABLE 3:**
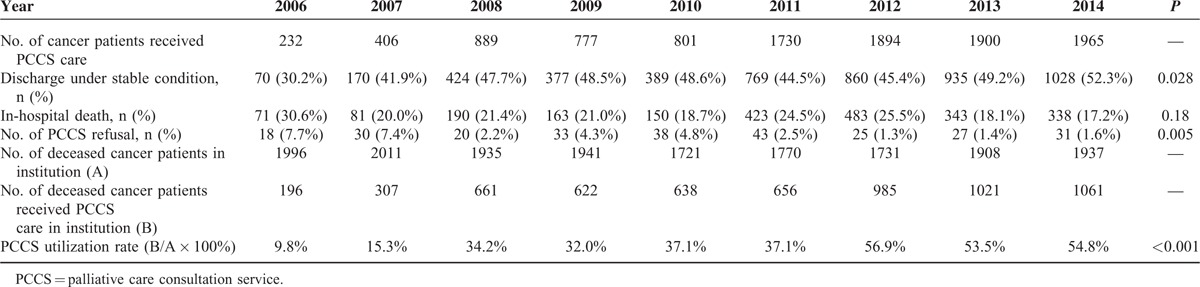
Patients’ Outcomes After Palliative Consultation Care Service (PCCS) Care

## DISCUSSION

Upward trends in PCCS utilization, patients’ and their family's disease awareness and DNR acceptance were observed at the beginning and the end of PCCS among terminally ill cancer patients in our institute between 2006 and 2014. However, a decrease tendency of PCCS refusal was also noticed during the same study period. The promotion of PCCS could increase patients and their family's disease awareness as well as terminally ill cancer patients’ DNR acceptance in Taiwan.

The order of cancer types among patients referred to PCCS in this study was similar to the sequence in cancer-related deaths in Taiwan since 2008, in addition, the numbers of patients referred to PCCS increased dramatically since 2008. The promotion of PCCS in our institute mainly from the strong endorsement of the oncologic department. Oncology department was the primary source of PCCS referral in this study, and we attributed this phenomenon to the following 2 reasons. First, patients treated at oncology department were more advanced and had shorter life span. Second, oncologists are more aware of the rights and end-of-life care for terminal ill cancer patients from their medical trainings.^15,16^ Our previous study showed that patients referred from oncology department had high disease awareness rates than those referred from other departments after PCCS.^13^ Another study also reported that cancer patients under the care of medical oncologist had fewer emergency department visiting, fewer intensive care unit admissions and were less likely to undergo cardiopulmonary resuscitation in the last month of their lives.^16^ Other specialists may be reluctant to refer patients to PCCS due to their lack of adequate specialty training,^17^ or feel uncomfortable to discuss issues regarding end-of-life care with patients and their family.^18–20^ The PCCS not only provides end-of-life care for patients and their family, but also provides a platform to interchange end-of-life care experience between primary care physicians and PCCS staffs. Our study showed that more and more patients were referred to PCCS by all departments in order to provide appropriate end-of-life care through mutual learning and collaboration between palliative care specialists and other specialists by means of the promotion of PCCS.

There was a trend of increasing both patients’ and their family's disease awareness starting from 2006, which coincide with the implementation of PCCS. Since 2005, PCCS was supported by a pilot program by the Bureau of Health Promotion in Taiwan. Our team was established in 2006 and provided services in the acute care setting and made available to terminal cancer patients. Our previous study showed that PCCS team can serve as a bridge to convey consensus from the patients and their family to the primary care staff resulted in improved quality of end-of-life care.^13^ With cumulated and shared experiences, PCCS team helped primary care professionals to learn crucial palliative care skills, such as explaining disease status and prognosis to both patients and their family, as well as discussing about the details of quality end-of-life care.^4,13^

In our study, families’ disease awareness was significantly higher than patients’ both at the beginning and the end of PCCS care. The differences of disease awareness between family and patients at the beginning and the end of PCCS care are presented in Figure 2. The predominant role of families overrode patients’ awareness of disease and prognosis was a unique situation in Taiwanese.^21^ In Taiwanese culture, families’ decisions predominate over patients’ opinions and implicit answers are preferred when breaking bad news. Therefore, the information regarding patients’ disease and prognosis were frequently modified according to their families’ requests. Although the differences of disease awareness between patients and their families decreased gradually at the end of PCCS care from 2006 to 2014, there was still around 30% of disease awareness between patients and their family in 2014. Hospice consultation service promotion could thus increase disease and prognosis awareness among patients and their families.^13^ However, to improve the differences of awareness between patients and their families, further continuous education for general population is required.

A DNR order increases quality of end-of-life care by avoiding unnecessary cardiopulmonary resuscitation in terminal cancer patients. In Taiwan, DNR designation for terminally ill patients was vigorously promoted by national policies after the enactment of Hospice Palliative Care Act in 2000. A nationwide survey conducted by the Taiwan National Health Insurance revealed a 58% decline in the rate of receiving resuscitation among cancer patients from 1997 to 2004, and the most significant decline occurred in 2000 after implementation of Hospice Palliative Care Act.^22^ Another population-based study from 2001 to 2006, involving 204,850 cancer decedents in Taiwan, showed that resuscitation rates substantially declined from 13.2% to 8.6%.^23^ A significant increase in DNR designation after PCCS care has been reported in patients with cancers or non-cancer diseases.^5^ Our previous study demonstrated that the effect of PCCS on DNR designation mainly resulted from increased patients’/families’ disease awareness and a longer duration of PCCS care.^24^ To achieve a better assessment of DNR designation probabilities, medical personnel should refer patients to the PCCS earlier and for longer durations, and promote patients’/families’ awareness of cancer prognosis.

In 2014, more than half (54.8%) of the cancer patients received palliative care in their last year of life in our institute. The percentage of PCCS utilization was increased 5-fold during the 9-year period after the promotion of PCCS. One nationwide survey reported in era without PCCS indicated that only 7.3% to 16.8% of Taiwanese cancer patients has used hospice services as their end-of-life care between 2000 and 2006.^3^ The promotion of PCCS solved the hospice demands of terminally ill patients who were reluctant to be transferred to an acute palliative unit, while the acute palliative unit was unavailable, or those whose conditions were too sick to be transferred.

Early palliative care referrals may improve patients’ care and quality of life by providing comprehensive physical, psychosocial, and spiritual distress evaluation and management. One recent study reported that patients with newly diagnosed metastatic nonsmall-cell lung cancer, who received standard oncology care integrated with early palliative care, were found to have a better quality of life and longer survival times.^25^ The observations from our study indicated that an increased percentage of patients were discharged under stable conditions and a decreased percentage of in-hospital deaths after PCCS care between 2006 and 2014. The results were attributable to the trend toward early palliative care referral after PCCS promotion.

The strength of our study resulted from its large sample size with a long recruitment period from a single medical center in Taiwan. However, limitations are as follows: First, this is a retrospective study analyzes data from years of clinical practice. Second, the impact of terminal cancer patients on end-of-life care were multifactored, this study was unable to address the significance of these factors other than PCCS. Furthermore, the associations between palliative care and quality of life as well as satisfaction with palliative care were not explored in our study.

## CONCLUSIONS

Our report briefly described the impact of PCCS on terminally ill cancer patients in a single medical center in Taiwan for a 9-year period. In the program of PCCS promotion, an increasing trend of palliative care consultation service utilization, better patients’ and their families’ awareness of diagnosis and prognosis, more consent to DNR, more patients were discharged with stable condition at the end of PCCS and a decrease refusal rate of end-of-life palliative care among terminal cancer patients were observed in Taiwan between 2006 and 2014.
